# GOParGenPy: a high throughput method to generate Gene Ontology data matrices

**DOI:** 10.1186/1471-2105-14-242

**Published:** 2013-08-08

**Authors:** Ajay Anand Kumar, Liisa Holm, Petri Toronen

**Affiliations:** 1 Institute of Biotechnology, University of Helsinki, (Viikinkaari 5), PO Box 56, Helsinki 00014, Finland; 2 Department of Biosciences, Division of Genetics, University of Helsinki, (Viikinkaari 5), PO Box 56, Helsinki 00014, Finland

**Keywords:** Gene Ontology, Large-scale datasets, Data Mining, Machine learning, Bioinformatics

## Abstract

**Background:**

Gene Ontology (GO) is a popular standard in the annotation of gene products and provides information related to genes across all species. The structure of GO is dynamic and is updated on a daily basis. However, the popular existing methods use outdated versions of GO. Moreover, these tools are slow to process large datasets consisting of more than 20,000 genes.

**Results:**

We have developed GOParGenPy, a platform independent software tool to generate the binary data matrix showing the GO class membership, including parental classes, of a set of GO annotated genes. GOParGenPy is at least an order of magnitude faster than popular tools for Gene Ontology analysis and it can handle larger datasets than the existing tools. It can use any available version of the GO structure and allows the user to select the source of GO annotation. GO structure selection is critical for analysis, as we show that GO classes have rapid turnover between different GO structure releases.

**Conclusions:**

GOParGenPy is an easy to use software tool which can generate sparse or full binary matrices from GO annotated gene sets. The obtained binary matrix can then be used with any analysis environment and with any analysis methods.

## Background

Gene Ontology (GO) is a popular standard in the annotation of gene products, providing information related to genes across all species. It presents a shared, controlled structured vocabulary of terms that describe the gene products [1]. GO is structured as a Directed Acyclic Graph (DAG) that holds the terms that describe the molecular function, biological process, and cellular component for a gene product. GO has a hierarchical structure that represents the terms from more specific to general terms.

GO is currently being used for various analysis tasks like a) over-representation of the GO classes from a selected group of genes [2], b) semantic similarity between two genes [3], c) threshold free gene set analysis [4,5], d) machine learning to classify unknown genes to various GO categories [6,7], and e) explorative analysis of large-scale datasets [8]. The linking of the reported GO categories to the GO DAG structure and their parent nodes is critical for all these tasks. In tasks a, c, d, and e, the parental nodes of the GO structure provide different levels of detail allowing simultaneous monitoring of very detailed and very broad functional classes. In the case of task b, the link to the GO hierarchy is crucial for finding a path between the two genes across the GO graph.

There are many existing methods [9-13] freely available for processing (i.e. linking gene products to the GO hierarchy) and analyzing Gene Ontology terms. Most of these tools perform well enough to handle small data sets, but on larger scale, such as in the case of microarray data, the execution time for these tools becomes prohibitive. Moreover, most of these methods use quite old GO structures causing methods to miss a large proportion of the currently used GO classes (see Results).

The annotationDbi [10] and GO.db [11] packages in Bioconductor are the most widely used tools for Gene Ontology analysis for the R enviroment. GO.db stores links from GO classes to their parent GO classes, storing all the GO classes, their parents, child terms and ancestor terms in a database for easy retrieval and processing. Despite the Gene Ontology consortium updating GO class annotations and linkages on daily basis, these GO related R packages are updated only biannually. Indeed, the best source of GO information is the annotation files themselves, which are available from the GO consortium web pages.

Even the GO consortium cannot help with research carried out with novel species. This is critical as we can expect a growing number of novel sequenced species with next-generation sequencing methods. These will require in-house GO annotation of sequences [7]. Also, with the analysis of more exotic organisms, there might be alternative sources for GO annotations, like species specific databases. Current GO processing tools use only a pre-fixed annotation source for analysis.

We present a fast python program named GOParGenPy (GO Parent Generation Python) that can process large annotation files, incorporate any version of OBO structure and can generate GO data matrices. Users of GOParGenPy will mainly be biologists and bioinformaticians who do analysis using languages, such as R, Matlab or python. It is freely available from the project web page (see Availability and requirements).

## Implementation

GOParGenPy has been implemented in Python (version requirement 2.5/2.6) and it is freely available as a standalone tool suitable for any downstream analysis related to GO data across various computing platforms. GOParGenPy generates the binary data matrix from a set of genes with GO annotation. It allows the user to select the GO annotation and the OBO structure file. The obtained GO binary matrix can then be used with any available analysis environment and with any available analysis methods. The main features of GOParGenPy are:

1. Reading in ‘gene_ontology_edit.obo’ file in standard format, parsing it and storing all the GO classes and their attributes.

2. Reading in the GO annotations of the analyzed genes (various input formats are supported).

3. Links GO annotations to their parent GO classes. The linking also looks for alternative ids for those GO classes which have become obsolete.

4. Outputs a list of genes with added parent GO classes.

5. Outputs a sparse or full matrix with genes as rows and GO classes as columns. The default format is the sparse matrix.

**Figure 1:**
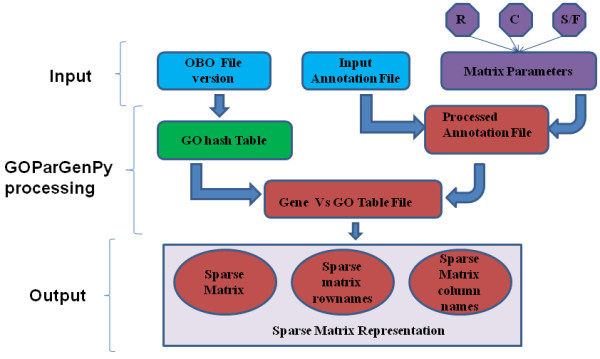
**Work flow of GOParGenPy.** Matrix parameters are row names (R, column number(s) for the input data column(s) where the gene names are reported), column names (C, column number(s) for the input data column(s) where the GO class associations are reported), and sparse or full matrix format (S/F).

Figure 1 shows the workflow of GOParGenPy. It takes in a tab separated input annotation file that contains a list of GO annotated genes, the selected OBO file and a set of parameters. These parameters denote the column number of gene name and the column number(s) of linked GO classes. Depending on the input annotation file type, an intermediate tab-delimited annotation file is then parsed from the annotation file where one row represents the gene name and all the collected GO annotations of this gene.

The OBO flat file format stores GO classes and attributes such as *id*, *name, namespaces, definition,* etc. OBO file GO classes and their respective attribute values are stored in a hash table using the numeric part of GO id as keys. Hence, the parent or ancestor class(es) for any given GO class can be retrieved recursively by looking through the attribute values of GO classes, namely ‘*is_a*’, ‘*part_of*’ and ‘*consider*’ links.

Next, the intermediate file obtained in first step is iterated over so that for each gene and its respective GO classes, all shared parent or ancestor GO classes are retrieved recursively using the above hash table. Redundant steps are removed by adding another hash table that is dynamically built as the iteration progresses through the entire file. The main purpose of this hash table is to store the GO class and all its parent or ancestor classes together so that when the same GO class is encountered in further iterations the retrieval does not get referred back to earlier GO hash table. Thus, at any instance the maximum size this data structure is the total number of GO classes present in a given OBO file. Hence, after certain stage the overall processing of input annotation file becomes independent of number of genes and the associated GO annotations.

Moreover, the program also does a lookup in the OBO file of alternate ids for any GO class which has become obsolete in order to retrieve parent/ancestor classes also in these cases. This functionality is optional.

**Figure 2:**
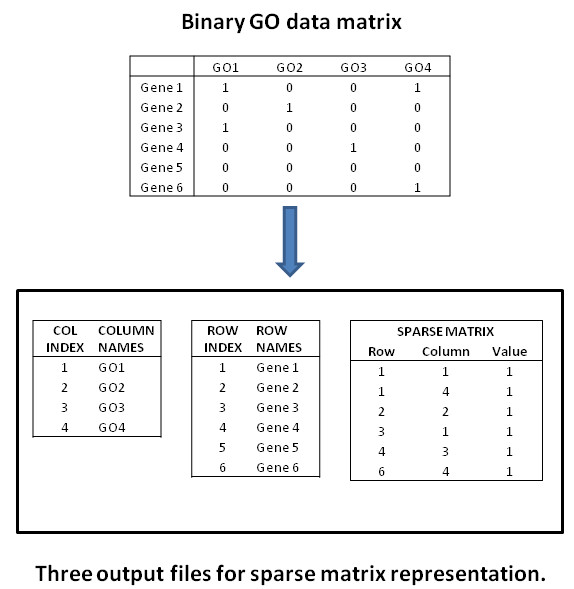
**Generation of sparse matrix with toy data.** Figure shows how a normal full matrix is converted to a sparse matrix. For each non-zero entry in the original matrix the sparse matrix stores three values: The row index, the column index and the value in the cell. GOParGenPy also reports the column and row names in separate files. Here the name tables include also indexing values for clarity.

Finally, user can specify whether a sparse or full binary matrix is generated with genes as row names and GO classes as column names. Reported GO classes are those occurring in the input annotation file and their parent nodes. Selection of the sparse matrix option is highly recommended as the package is intended for large datasets (>20,000 GO annotated genes). Sparse matrices are memory efficient representations for matrices where most of the values are zero. This is the case with GO data matrices as large part of GO classes have less than one percent of genes as members and the non-members are given value zero. We use the sparse matrix representation with three columns. These columns represent the row number and column number of non-zero value and the value in the cell. Figure 2 demonstrates this process.

The obtained sparse matrix can be further processed with standard analysis pipelines. The sparse matrix format is supported by many analysis environments, like R and Matlab.

## Methods

We compare GOParGenPy against existing methods (DAVID [12], agriGO [13], AnnotationDBI and GO.db from R/Bioconductor and GeneOntology package in Bioperl Toolkit [14]) using two metrics:

1. Instability of OBO files.

2. Execution time.

### Instability of OBO files

OBO files are central to all GO analysis. However, they vary significantly between GO analysis tools with DAVID using version 6.7, agriGO using version 1.2 and GO.db/AnnotationDBI from R/Bioconductor using a biannually updated version.

Therefore, we highlight the benefits of GOParGenPy’s ability to allow selection of any OBO structure by showing the information loss when an older OBO structure is used instead of the latest structure. Here the aim is to find what percentage of current GO classes is missing in these older OBO packages. Hence, respective OBO version corresponding to last update of these packages is downloaded from the GO website. The versions are:

1. For DAVID the corresponding version of OBO file used is of date 01.12.2009

2. For GO.db the corresponding version of OBO file used is of date 01.03.2011

3. For agriGO the corresponding version of OBO file used is of date 01.04.2010

4. The reference version of OBO file with which these packages are compared is of date 01.02.2012.

**Figure 3:**
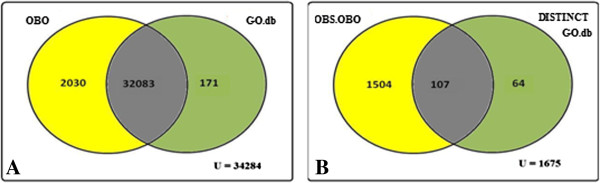
**Venn diagram representation of total number of GO classes present in OBO file ‘gene_ontology_edit.obo’ (01.02.2012) and “GO.db” package version 2.5 (01.03.2011) of R/Bioconductor.** Figure 3**A:** Venn representation of number of non obsolete GO classes from OBO file and GO.db package. Figure 3**B:** Venn representation of number of obsolete GO classes in OBO file and distinct non obsolete GO classes in GO.db.

**Figure 4:**
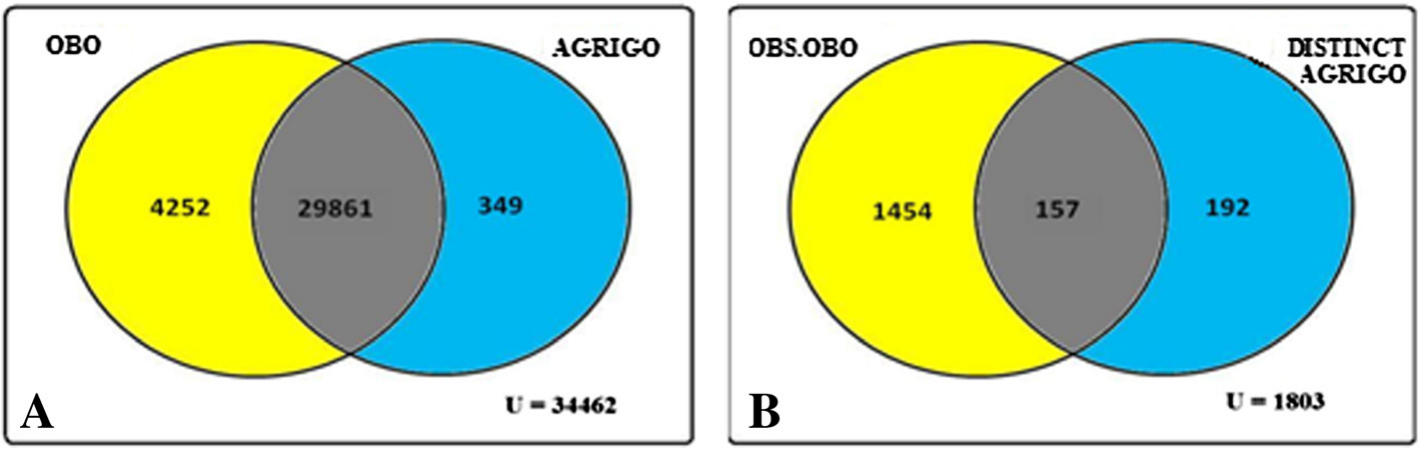
**Venn diagram representation of total number of GO classes present in OBO file ‘gene_ontology_edit.obo’ (01.02.2012) and OBO file (01.04.2010) from agriGO.** Figure 4**A:** Venn representation of number of non obsolete GO classes from OBO file and agriGO. Figure 4**B:** Venn representation of number of obsolete GO classes in OBO file and distinct non obsolete GO classes in agriGO.

**Figure 5:**
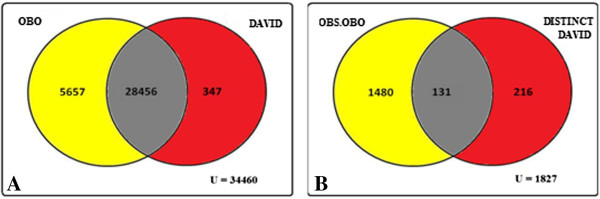
**Venn diagram representation of total number of GO classes present in OBO file ‘gene_ontology_edit.obo’ (01.02.2012) and OBO file (01.12.2009) from DAVID.** Figure 5**A:** Venn representation of number of non obsolete GO classes from OBO file and DAVID. Figure 5**B:** Venn representation of number of obsolete GO classes in OBO file and distinct non obsolete GO classes in DAVID.

These files were parsed for GO classes using GOParGenPy. Next we calculated 1) the number of actual GO classes with unaltered definitions, 2) the number of GO classes which became obsolete and 3) the number of GO classes that have an altered definition with respect to the reference OBO file. Finally, we present a Venn diagram to show the percentage of missing GO classes and actual classes present (Figures 3, 4, 5 in Results).

### Relative execution time

The execution time was compared only between the most widely used standalone packages. These are GeneOntology package from Bioperl Toolkit, GO.db and AnnotationDBI from R/Bioconductor. The aim is to compare the performance of GOParGenPy with these packages in processing large datasets. Parent GO classes were generated by GOParGenPy using the current version of GO structure (01.03.2013). First, the methods were tested with a randomly chosen set of 80975 genes from UNIPROT-GOA [15]. This is 2–3 times the size of the largest genomes in gene expression analysis. Next, in order to measure the performance on extremely large file, tools were tested with all the GO annotated sequences (>21 million sequences) available from UNIPROT-GOA.

## Results

### Comparative analysis of GO packages

The comparative analysis details for AnnotationDBI/GO.db, agriGO, DAVID, are shown in Figures 3, 4, and 5 respectively.

It is evident from above figures that OBO structures in the evaluated GO web tools GO.db, agriGO and DAVID miss a significant number of currently used GO classes. In Figure 3A, the total number of non-obsolete distinct GO classes from OBO file is 2030 and 171 distinct non-obsolete GO classes in GO.db. Subsequently, in Figure 3B, 107 of these non-obsolete GO classes have their definition as obsolete with respect to the reference version (01.02.2012) of OBO file. Thus, in total 3641 (2030+1611) GO classes has been added or their definition has been altered with respect to non-obsolete GO classes of GO.db package (01.03.2011). This corresponds to 11.30% of GO classes being altered. Similarly, from Figure 4A,B, it can be seen that the total number of non-obsolete distinct GO classes from OBO file is 4252 and 349 distinct non-obsolete GO classes in agriGO. Correspondingly, 157 of these GO classes in agriGO have their definition altered with respect to current version of OBO file. Together, it can be seen that 19.40% of GO classes are altered. Finally, from Figure 5A,B it can be observed that in DAVID a total of 25.2% of GO classes have been altered. From Table 1, it can be found that on average 2572 GO classes are altered each year. This shows the level of change in the number of GO classes and clearly indicates the importance of using the current version of OBO structure.

**Table 1:** A comparative view of change in total number of GO classes per year throughout 9 years of OBO files data

**Year**	**Total GO Classes**	**Change in number of GO**	**Average change in GO**
		**classes per year**	**classes in 8 years**
**2004**	18219	-	2572
**2005**	20349	2130	
**2006**	22929	2580	
**2007**	25771	2842	
**2008**	27867	2096	
**2009**	30716	2849	
**2010**	33268	2552	
**2011**	35724	2456	
**2012**	38794	3070	

### Execution time

Table 2 compares the running time between GOParGenPy, GO.db/AnnotationDbi from Bioconductor and GeneOntology package from Bioperl toolkit. With set of 80975 GO annotated genes GOParGenPy took approximately 40 seconds to generate data matrices making it almost 10 times faster than competing methods. GeneOntology package from Bioperl was relatively closer to GOParGenPy’s execution time but BioPerl only performed the mapping of annotated genes to parent nodes and did not print any output file, whereas GOParGenPy also generated the output files. With large data set consisting over 21 million sequences from UNIPROT-KB the competing methods were unable to finish in a reasonable time. Although this dataset size is outside the standard analysis requirements, it gives a good extreme performance test.

**Table 2:** Execution time of various available packages

**Number of Genes**	**AnnotationDbi/GO.db Package in**	**GeneOntology package in**	**GOParGenPy**
	**R/Bioconductor**	**BIOPERL**	
**80975**	>592* sec	>300* sec	40 sec
**21318390**	d.n.f^#^	d.n.f^#^	3300 sec

Tests were performed on a 2.93GHz Intel Xeon X7350 with 64 GB RAM. Note that only GOParGenPy created also the output files in these tests, whereas the other packages only linked the annotations to the parent classes.

^*^indicates the time taken by these packages only to generate the parent GO classes for given GO classes associated with these genes.^#^*execution time was too long. d.n.f: did not finish.*

## Discussion

We present a new standalone software tool GOParGenPy for generating high-throughput GO data matrices for any selected input annotation file and any version of OBO file. We have shown the importance of OBO structure and presented an effective way of storing and retrieving GO classes and their attributes for any downstream analysis involving GO data. All the existing methods, be it web based application or standalone offline tools, utilize an outdated OBO structure from GO consortium. As shown in the Figures 3 – 5 we can find that at maximum 25% of GO classes (for DAVID tool) are outdated with respect to current version of OBO file. Hence, any downstream analysis methods that incorporate GO data obtained from these tools may lead to erroneous results.

GOParGenPy outperforms all these existing tools in terms of incorporating users’ choice of OBO structure and speed of generating GO data matrices. It is also able to process extremely large datasets. It incorporates a dynamic hash table that stores all GO classes from the input file with their parent GO classes retrieved from OBO structure. This unique feature enables generation of data matrices independent of size of input data as the maximum size of this hash table is the total number of GO classes present in the OBO structure file used. Hence, this makes GOParGenPy faster in the generation of GO data matrices for large gene sets. Also, GOParGenPy looks for alternative ids of those GO classes which have become obsolete or have their definition altered.

Although, GOParGenPy does not do any actual data analysis or visualization steps itself the output files can be easily imported to environments like Matlab, R or Python. The output GO data can be used as an input for various analysis tasks like prediction of new GO annotations with classifiers [6], for visualization tasks [8] or for correlation analysis between GO data and large-scale data [3]. Thus, GOParGenPy encourages modular thinking in bioinformatics.

GOParGenPy allows the user to select the used GO annotation file and the used GO structure file. This allows the usage of latest annotation data files and latest GO structure. However, it can also be used with older annotation files. This is useful when an older work needs to be replicated, or while comparing methods with one that uses old GO structure.

Additionally, GOParGenPy features and its application can be extended to other ontology resources and it has been already tested with Plant Ontology (PO). GOParGenPy optional features can incorporate any PO annotated gene lists and corresponding OBO file to generate sparse binary matrix representation. (see Project homepage).

## Conclusions

GOParGenPy is a fast python program for generating GO binary data matrices from annotated set of genes. GOParGenPy outperforms existing tools by allowing any available version of the OBO structure and handling large scale input annotation dataset with over 21 million annotated sequences. The output files can be easily incorporated into various platforms such as MATLAB, R or Python for further GO related downstream analysis.

## Availability and requirements

**Project name:** GOParGenPy

**Project homepage:**http://ekhidna.biocenter.helsinki.fi/users/ajay/private/GOParGenPy.htm

**Operating system:** Platform independent

**Programming language:** Python version 2.5/2.6

**License:** Free for academic use

## Competing interests

The authors declare that they have no competing interests.

## Author’s contributions

PT described the problem statement. AAK developed and implemented the software tool. LH and PT supervised the project. All authors contributed equally in writing the manuscript. All authors read and approved the final manuscript.
